# Psychometric properties of the Zephyr bioharness device: a systematic review

**DOI:** 10.1186/s13102-018-0094-4

**Published:** 2018-02-21

**Authors:** Goris Nazari, Pavlos Bobos, Joy C. MacDermid, Kathryn E. Sinden, Julie Richardson, Ada Tang

**Affiliations:** 1 Physical Therapy, Western University, London, ON Canada; 2 Roth McFarlane Hand and Upper Limb Centre, St. Joseph’s Hospital, London, ON Canada; 3 School of Kinesiology, Lakehead University, 955 Oliver Road, Thunder Bay, ON Canada; 4 McMaster University, School of Rehabilitation Science, 1400 Main Street West, Hamilton, ON Canada

**Keywords:** Zephyr bioharness, Psychometric properties, Reliability, Validity, Agreement, Wearable device, Heart rate, Quality evidence

## Abstract

**Background:**

Technological development and improvements in Wearable Physiological Monitoring devices, have facilitated the wireless and continuous field-based monitoring/capturing of physiologic measures in healthy, clinical or athletic populations. These devices have many applications for prevention and rehabilitation of musculoskeletal disorders, assuming reliable and valid data is collected. The purpose of this study was to appraise the quality and synthesize findings from published studies on psychometric properties of heart rate measurements taken with the Zephyr Bioharness device.

**Methods:**

We searched the Embase, Medline, PsycInfo, PuMed and Google Scholar databases to identify articles. Articles were appraised for quality using a structured clinical measurement specific appraisal tool. Two raters evaluated the quality and conducted data extraction. We extracted data on the reliability (intra-class correlation coefficients and standard error of measurement) and validity measures (Pearson/Spearman’s correlation coefficients) along with mean differences. Agreement parameters were summarised by the average biases and 95% limits of agreement.

**Results:**

A total of ten studies were included: quality ratings ranged from 54 to 92%. The intra-class correlation coefficients reported ranged from 0.85–0.98. The construct validity coefficients compared against gold standard calibrations or other commercially used devices, ranged from 0.74–0.99 and 0.67–0.98 respectively. Zephyr Bioharness agreement error ranged from − 4.81 (under-estimation) to 3.00 (over-estimation) beats per minute, with varying 95% limits of agreement, when compared with gold standard measures.

**Conclusion:**

Good to excellent quality evidence from ten studies suggested that the Zephyr Bioharness device can provide reliable and valid measurements of heart rate across multiple contexts, and that it displayed good agreements vs. gold standard comparators – supporting criterion validity.

**Electronic supplementary material:**

The online version of this article (10.1186/s13102-018-0094-4) contains supplementary material, which is available to authorized users.

## Background

Technological development and improvements in Wearable Physiological Monitoring (WPM) devices, have facilitated the wireless, long range and continuous field-based monitoring/capturing of physiologic measures in healthy, clinical or athletic populations [1–3]. Numerous WPM devices have been introduced to the market [4, 5] with a range of capabilities and target audiences.

The Zephyr Bioharness ™ (Zephyr Technology Corporation, Annapolis, MD, US) is a wireless chest-based wearable device, capable of real-time and long-distance recording of various physiological parameters, including heart rate, respiratory rate, core temperature, activity levels and posture [6]. The device can capture data for 26 h, includes a BioModule, weighs 85 g and fits on the chest at lower sternum for both men and women [6]. The BioModule is snapped into an adjustable belt. The belt (chest strap) contains skin conductive electrodes to captures heart rate through recording of cardiac electric impulses, and produces an output in beats per minute. Heart rate monitoring offers several advantages. Calculating the percentage of maximum heart rate, is a commonly used approach to monitor exercise intensity. [7]. Among athletes (soccer players), submaximal exercises heart rate monitoring has shown to be highly predictive of improvements in physical performance (i.e. maximal aerobic speed) [8]. In addition, during steady-state exercise, the linear relationship between heart rate and the rate of oxygen consumption has shown to be an effective method to assess training internal load [9]. This linear relationship can also be used to estimate maximal oxygen uptake VO2max [10]. Furthermore, in both trained individuals and athletes, monitoring of heart rate recovery has been suggested as a potential marker to evaluate training status, which is, in turn, used to optimize training programs [11]. It has been proposed that heart rate measures can be used to provide an estimate of energy expenditure, providing an easier and inexpensive alternative [12].

It is important for a device to be reliable (provide consistent scores in stable conditions), valid (provide true scores) and be responsive (to detect change over time) if it is to be used to assess/support performance or decision-making [13, 14]. Reliability is measured in both relative and absolute terms. Relative reliability – a correlation coefficient, comments on the ability of a device to differentiate between participants, whereas absolute reliability emphasizes on the measurement error in the same unit of original measurement [13]. In order for a device to be useful (reliable), its relative reliability needs to be sufficiently large, and absolute reliability sufficiently small [13]. A device can be reliable but not valid [13]. Validity can be assessed in a variety of ways, but ideally is established by comparing devices to an established “gold standard” criterion measure, with criterion validity established when a new device can provide the same measurement as the standard [15]. In addition, neither the reliability nor the validity measurement properties of a device can be used to detect change over time (improvements or deteriorations). Reporting of the responsiveness parameters of a device, deals with its ability to assess change over time [13, 14].

Individual measurement studies often address some domains of measurement, but do not provide comprehensive assessments [16–18]. Systematic reviews of measurement studies allow for one to understand the measurement properties across a variety of contexts, populations and measurement purposes. By using a structured clinical measurement specific appraisal tool, we are able to focus on higher quality research when synthesizing measurement research [16–18].

Considering heart rate and its wide-spread application, and the need to synthesis and provide a comprehensive evidence on the accumulating measurement properties of Zephyr Bioharness device, the aims of this systematic review were to synthesize and critically appraise the measurement studies where a Zephyr Bioharness device was used to measure heart rate.

## Methods

### Search

To identify articles on psychometric properties of Zephyr Bioharness device, we searched the Embase, Medline, PsycInfo, PuMed and Google Scholar databases between January 2010 – January 2017, using the following keywords: Zephyr Bioharness OR ZB) AND (heart rate OR psychometric properties OR measurement properties OR reliability OR minimal detectable change OR validity OR responsiveness OR minimal clinical important difference OR agreement. Further articles were also identified by examining the reference list of each selected study. We were specifically interested in Zephyr Bioharness device, which has been introduced into the market at year 2010, so we limited our search to this year because we did not any expect publications prior to that year.

### Selection of studies

At the first stage, two authors independently identified and screened Title/abstract. Studies that had used the device to monitor physiological measures only, without reporting of psychometric properties were considered irrelevant. An article was accepted if it met following specific eligibility criteria:

Inclusion Criteria:

1. Purpose of the study states assessing reliability or validity or responsiveness or agreement parameters, of Zephyr Bioharness heart rate variable in healthy or clinical population.
2. Articles published in English,

Exclusion Criteria:

1. No data on the psychometric properties of Zephyr Bioharness heart rate variable.
2. Studies that had used Zephyr Bioharness device to monitor physiological responses only.

### Data extraction

The primary author G. N., and secondary author P. B. conducted the data extractions. For reliability measures, Standard Error of Measurement (SEM), intra-class correlation coefficient (ICC), mean differences and confidence intervals were extracted [16–18]. These were interpreted using a common benchmark where ICC < 0.40 indicate poor, 0.40 ≤ ICC < 0.75 indicate fair to good and ICC ≥ 0.75 indicate excellent reliability [19]. For construct validity where these devices were compared against a reference standard, Pearson’s/Spearman’s correlation coefficients and mean difference data were extracted [16–18]. The absolute value for the strength of the correlation were determined using the guide suggested by Evans [20] as follows; 0.00–0.19 “very weak”, 0.20–0.39 “weak”, 0.40–0.59 “moderate”, 0.60–0.79 “strong”, 0.80–1.00 “very strong”. To assess levels of agreement, agreement bias along with 95% Limits of Agreement (LoA) were extracted. This uniquely evaluates whether there is a discrepancy (bias) between two different devices measuring the same construct [21].

### Quality appraisal

The articles were appraised by the first (G. N.) and second (P. B.) authors for quality using a structured clinical measurement specific appraisal tool [16–18]. This quality tool has previously demonstrated high reliability in evaluating the quality of clinical measurement studies for musculoskeletal outcome measures [18]. The evaluation criteria included: 1) Thorough literature review to define the research question; 2) Specific inclusion/exclusion criteria; 3) Specific hypotheses; 4) Appropriate scope of psychometric properties; 5) Sample size; 6) Follow-up; 7) The authors referenced specific procedures for administration, scoring, and interpretation of procedures; 8) Measurement techniques were standardized; 9) Data were presented for each hypothesis; 10) Appropriate statistics-point estimates; 11) Appropriate statistical error estimates; and 12) Valid conclusions and recommendations [16–18] (Additional file 1). An article’s total quality score was calculated by summing of scores for each item, divided by the numbers of items and multiplied by 100% [16–18]. Quality summary of appraised papers that ranged from (0%–30%) was marked as Poor, (31%–50%) as Fair, (51%–70%) as Good, (71%–90%) as Very Good, and (> 90%) as Excellent [16, 17]. When individual appraisals varied, we used the below consensus procedures:

1. We first identified the variations in individual appraisals.
2. To resolve scoring discrepancies based on factual content, the original articles were reassessed.
3. To resolve scoring discrepancies based on extent of compliance with the item, the raters (G.N.) and (P.B.) consulted the third author/instrument developer (J.M.).
4. The first and the second authors further discussed their understanding of how well articles complied with each item of the appraisal tool. The most common source of score discrepancies were oversight.

## Results

**Fig. 1:**
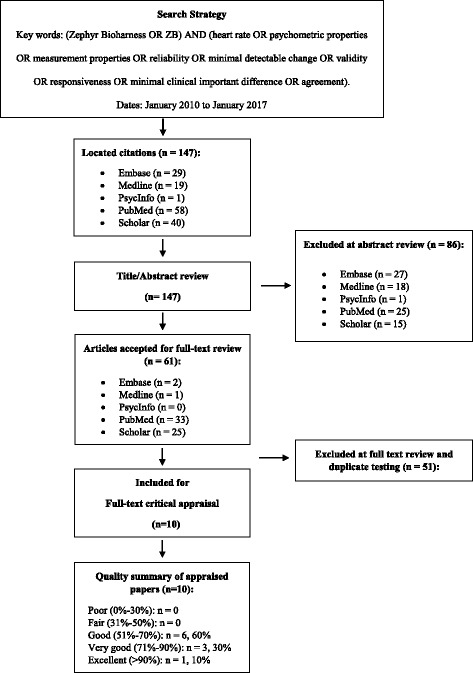
The systematic review evidence flowchart

A total of 147 studies were identified from the search in the databases [Embase (*n* = 29), Medline (*n* = 19), PsycInfo (n = 1), PubMed (*n* = 58) and Google Scholar (*n* = 40)], of which 61 studies were considered relevant. All 61 studies were retrieved and assessed for eligibility, and a total of 10 studies were included in this review (Fig. 1). Table 1 displays the summary of the studies addressing the psychometrics of Zephyr Bioharness device. The quality of the studies ranged from 54 to 92%, with 80% of articles reaching or exceeding a score of 67% on the quality rating (Fig. 1 & Table 2). The most common flaws noted in were 1) lack of specific hypotheses, 2) not considering an appropriate scope of psychometric properties/ lack of specific inclusion or exclusion criteria, and 3) lack of a sample size calculation/justification.

**Table 1:** Summary of studies addressing psychometric properties of ZB

Study	Sample	n	Properties evaluated	Testing protocol
Johnstone et al. (2012). [1]	Ten physically active males, age 21.5 ± 2.8 years, weight 71.4 ± 7.9 kg and height 1.79 ± 0.1 m.	10	Reliability, concurrent validity & agreement.	Treadmill walk-jog-run.
Johnstone et al. (2012a). [24]	Twenty-two physically active males, age 21.5 ± 2.8 years, weight 71.4 ± 7.9 kg and height 1.79 ± 0.10 m.	22	Concurrent validity & agreement.	Treadmill walking and running.
Johnstone et al. (2012b). [2]	Ten physically active males, age 20.5 ± 2.1 years, weight 70.4 ± 9.4 kg and height 1.77 ± 0.10 m.	10	Reliability.	Treadmill running.
Kim et al. (2013). [25]	Twelve healthy men, age 25.5 ± 4.1, height 180.1 ± 6.5 and weight 78.8 ± 13.9.	12	Validity & agreement.	Treadmill running.
Gatti et al. (2014). [26]	Seven healthy males and three females, Age 23.8 ± 2.9 years, height 179 ± 8 cm and weight 75.5 ± 10.7 kg.	10	Validity & agreement.	Sitting, thoracic rotation, arm Lifting, Batting, Weight-moving, Walking.
Smith et al. (2014). [29]	Eleven healthy men age 20.0 ± 1.0 years, height 1.80 ± 0.07 m and weight 82.0 ± 10.2 kg.	11	Validity & agreement.	Treadmill walk, search (crawl), stairs.
Dolezal et al. (2014). [28]	Ten healthy men, age 21.0 ± 1.0 years, height 184 ± 5 cm and weight 91 ± 10 kg.	10	Criterion validity & agreement.	Treadmill walk, search (crawl), stairs, fast walk.
Flanagan et al. (2014). [27]	Seventy-five healthy men, age 23 ± 4 years, height 181 ± 8 cm and weight 83 ± 12 kg.	75	Concurrent validity & agreement.	Cycle ergometery (rest, 12-stages of cycle ergometer and recovery).
Rawstorn et al. (2015). [22].	Six recreationally active males and four females, Age 26.68 ± 3.26 years, weight 71.10 ± 11.53 kg and height 1.73 ± 0.06 m. Five males and three females with atrial fibrillation, Age 69.68 ± 9.53 years, weight 77.46 ± 18.81 kg and height 1.69 ± 0.12 m.	10 8	Reliability, validity & agreement.	Treadmill running. Treadmill, cycle ergometery and activities of daily living: sweep and vacuum.
Nazari et al. (2017). [23]	Thirty healthy males 48.0 ± 15 years, body mass index 25.0 ± 2.30 kg/m^2^, and 30 females 30 females, 48.0 ± 15 years, body mass index 24.0 ± 3.50.	60	Reliability	Rest, sub-maximal activity, recovery.

**Table 2:** Quality of studies on the psychometric properties oF ZB

Study	Item evaluation criteria ^a^												
	1	2	3	4	5	6	7	8	9	10	11	12	Total (%)
Nazari et al. (2017). [23]	2	2	2	1	2	2	2	2	2	2	1	2	92
Johnstone et al. (2012). [1]	2	1	1	1	0	2	2	2	2	2	2	2	79
Johnstone et al. (2012a). [24]	2	1	1	1	0	NA	2	2	1	2	2	2	73
Flanagan et al. (2014). [27]	1	1	1	1	1	NA	2	2	2	2	1	2	73
Smith et al. (2014). [29]	2	1	1	1	0	NA	2	2	1	2	2	1	68
Dolezal et al. (2014). [28]	1	1	1	1	0	NA	2	2	2	2	1	2	68
Kim et al. (2013). [25]	2	1	1	1	0	NA	2	2	1	2	1	1	64
Rawstorn et al. (2015). [22]	1	1	1	1	0	2	2	2	1	2	1	1	63
Johnstone et al. (2012b). [2]	2	1	1	0	0	0	2	2	1	2	1	2	58
Gatti et al. (2014). [26]	1	1	0	1	0	NA	2	2	1	2	1	1	54

Total score = (sum of subtotals ÷ 24 × 100). If for a specific paper an item is deemed NA (Not Applicable), then, Total score = (sum of subtotals ÷ (2 × number of Applicable items) × 100)

Quality Summary: Poor (0%–30%), Fair (31%–50%), Good (51%–70%), Very good (71%–90%), Excellent (> 90%):

*NA* not applicable. The subsections no. 6, asks for percentage of retention/follow up. This subsection only applies to reliability test-retest studies

^a^ Item Evaluation Criteria: 1. Thorough literature review to define the research question; 2. Specific inclusion/exclusion criteria; 3. Specific hypotheses; 4. Appropriate scope of psychometric properties; 5. Sample size; 6. Follow-up; 7. The authors referenced specific procedures for administration, scoring, and interpretation of procedures; 8. Measurement techniques were standardized; 9. Data were presented for each hypothesis; 10. Appropriate statistics-point estimates; 11. Appropriate statistical error estimates; 12. Valid conclusions and clinical recommendations

### Zephyr bioharness heart rate reliability

We located four studies that examined the test-retest reliability measures of Zephyr Bioharness [Table 3] during different physical activities including rest, recovery phases and unstructured mobility; vacuuming and sweeping, and structure running/walking, cycling and submaximal activity [1, 2, 22, 23]. The populations studied included young healthy recreational active males and females across various age groups as well as older patients with atrial fibrillation [1, 2, 22, 23].

**Table 3:** Summary of reliability properties of ZB

Study	Testing protocol	ICC	SEM/CV	Mean diff.	95% C.I.
Johnstone et al. (2012). [1]	Walk-Jog-Run				
	All Velocities	0.97	4.60 (CV)	4.30	−4.56 – 3.92
	4–6 km/h	0.89	5.90 (CV)	− 0.20	− 2.40 – − 1.23
	8–10.5 km/h	0.93	4.10 (CV)	5.10	−5.55 – − 4.71
	11.0 km/h	0.85	2.80 (CV)	5.60	−6.32 – − 4.82
Johnstone et al. (2012b). [2]	Treadmill Running	0.98	4.80 (CV)	2.70	−3.15 – 2.22
Rawstorn et al. (2015). [22]	Treadmill Running	0.98	5.20 (SEM)	–	–
	Treadmill, Cycle Ergometer and Activities of daily living; sweep and vacuum	0.98	4.77 (SEM)	–	–
Nazari et al. \(2017). [23]	Rest	0.92	2.11 (SEM)	0.21	−3.93 – 4.35
	Submaximal activity	0.94	3.50 (SEM)	2.82	−4.04 – 9.68
	Recovery	0.90	3.51 (SEM)	1.56	−5.31 – 8.43

*ZB* zephyr bioharness, *ICC* intra-class correlation coefficient, *SEM* standard error of measurement, *CV* coefficient of variation, *Mean diff* mean difference, *95% C.I.* confidence interval

Overall, ZB heart rate variable displayed excellent reliability properties. This included a SEM ranging from 2.11–5.90 beats per minute and, excellent test re-test reliability coefficients ≥0.85 [1, 2, 22, 23].

### Zephyr bioharness heart rate validity

We identified two studies that assessed the validity of ZB heart rate variable against other commercially used devices (Polar T31) [1, 24], and six studies that assessed validity against gold standard criterion measure (ECG) [Table 4] [22, 25–29]. Validity measures were established at resting, physical activity, and recovery phases, including both healthy recreational active males and females, as well as older patients with atrial fibrillation [1, 22, 24–29].

**Table 4:** Summary of validity properties of ZB

Study	Criterion measure	Testing protocol	Mean diff.	r/r_s_
Johnstone et al. (2012a). [24]	Polar-T31.	Treadmill – Walking and Running	−3.80	0.89
Johnstone et al. (2012). [1]	Polar-T31	Walk-Jog-Run		
		All Velocities	0.00	0.98
		4–6 km/h	1.30	0.92
		8–10.5 km/h	− 0.70	0.93
		11.0 km/h	−2.10	0.67
Kim et al. (2013). [25]	12-Lead ECG	Treadmill running	3.20	0.87
Flanagan et al. (2014). [27]	5 Lead ECG	Cycle ergometer		
		Rest	1.00	≥0.99
		12- Stages	0.00	≥0.99
		Recovery	2.00	≥0.99
Dolezal et al. (2014). [28]	12 Lead ECG	Treadmill Walk	0.04	0.99
		Search (Crawl)	− 0.01	0.99
		Stairs	−0.13	0.99
		Fast walk	0.03	0.99
Smith et al. (2014). [29]	3 Lead ECG	Treadmill Walk	−0.40	0.99
		Search (Crawl)	−1.70	0.95
		Stairs	0.40	0.99
Gatti et al. (2014). [26]	5 Lead EKG	Sitting	−0.78	0.99
		Thoracic rotation.	−0.77	0.98
		Arm Lifting.	0.22	0.94
		Batting.	−2.51	0.76
		Weight moving.	−4.81	0.78
		Walking.	−1.68	0.74
Rawstorn et al. (2015). [22]	12 Lead ECG	Treadmill Running	−1.30	0.92
		Treadmill, Cycle Ergometer and Activities of daily living; sweep and vacuum	−1.45	0.97

*ZB* zephyr bioharness, *Mean diff* mean difference, *r* Pearson’s correlation coefficient, *r*_*s*_ spearman’s rank correlation

In summary, the ZB displayed strong to very strong correlations of ≥0.67 during physical activity phases when compared with Polar T31 device [1, 24]. In addition, the device demonstrated very strong correlations of ≥0.87 at rest [25–27], strong to very strong correlations of ≥0.74 during various activities [22, 25–29] and very strong correlations of ≥0.99 throughout recovery [27] when compared with a gold standard criterion measure (ECG).

### Zephyr bioharness heart rate agreement

We identified two studies that assessed the pair-wise agreement between ZB heart rate measure with Polar T31 device [1, 24], and six studies that assessed the pair-wise agreement between ZB heart rate measure with gold standard criterion measure (ECG) [Table 5] [22, 25–29].

**Table 5:** Summary of agreement properties of ZB

Study	Criterion measure	Testing protocol	Agreement bias	95% LoA
Johnstone et al. (2012a). [24]	Polar-T31.	Treadmill – Walking and Running	−3.05	−32.20 – 32.20
Johnstone et al. (2012). [1]	Polar-T31	Walk-Jog-Run	0.02	−79.20 – 79.20
Kim et al. (2013). [25]	12-Lead ECG	Treadmill running	0.50	−15.30 – 16.30
Flanagan et al. (2014). [27]	5 Lead ECG	Cycle ergometer	3.00	−2.84 – 2.42
Dolezal et al. (2014). [28]	12 Lead ECG	Treadmill Walk	0.04	−0.05 – 0.12
		Search (Crawl)	−0.01	− 0.12 – 0.10
		Stairs	−0.13	−0.21 – − 0.04
		Fast walk	0.03	−0.09 – 0.14
Smith et al. (2014). [29]	3 Lead ECG	Treadmill Walk	− 0.40	−0.70 – − 0.10
		Search (Crawl)	−1.70	−3.1 – −0.40
		Stairs	0.40	0.04–0.70
Gatti et al. (2014). [26]	5 Lead EKG	Sitting	−0.78	−5.10 – 3.60
		Thoracic rotation.	−0.77	−5.40 – 3.90
		Arm Lifting.	0.22	−7.70 – 8.10
		Batting.	−2.51	−15.70 – 10.70
		Weight moving.	−4.81	−22.50 – 12.90
		Walking.	−1.68	−15.60 – 12.20
Rawstorn et al. (2015). [22]	12 Lead ECG	Treadmill Running	−0.30	−21.87 – 9.26
		Treadmill, Cycle Ergometer and Activities of daily living; sweep and vacuum	1.10	−13.39 – 23.79

*ZB* zephyr bioharness, *95% LoA* limits of agreement

Three studies reported heart rate biases of ≤3.00 beats per minute with (− 3.10–2.42) 95% limits of agreement in pairwise device comparison of ZB at rest, recovery phases or during various activities against ECG [27–29]. Furthermore, the inter-device agreement between ZB and Polar T31 heart rate measures yielded agreement biases of ≤3.05 with (− 79.20–79.20) 95% limits of agreement during a treadmill walk/run testing protocols [1, 24].

Overall, ZB heart rate variable displayed better agreements (i.e. narrower limits of agreement) with ECG than with Polar T31 device, supporting criterion validity and suggestive of possible interchangeable use [1, 22, 24–29].

## Discussion

After synthesizing ten studies addressing the measurement properties of the Zephyr Bioharness device, we conclude that there is good to excellent quality evidence supporting the reliability and validity of this device. This review suggests that the Zephyr Bioharness device can provide reliable and valid measurements of heart rate across multiple contexts, and that it might be useful for prevention or rehabilitation applications where field-based monitoring of heart rate is required in low risk patient populations. The use of the devices in high-risk populations was not studied.

In regards to ZB reliability parameters, four studies were identified [1, 2, 22, 23]. The included studies reported sufficiently large relative reliability scores, and sufficiently small absolute reliability measures. All four identified studies reported excellent ICC ≥ 0.85 (SEM ≤ 5.90) during various physical activities, and excellent ICC ≥ 0.90 (SEM ≤ 3.51) at resting and recovery phases [1, 2, 22, 23].

Validity coefficients quantify the linear relationship between two measures /devices [15]. However, the coefficients do not provide information regarding the extent of systematic error (lack of agreement) between two devices. Since it is very rare to obtained two identical findings while assessing the same construct using two different devices, reporting of the magnitude of the agreement is necessary [15]. Reporting of individual agreement in terms of 95% limits of agreement (LoA), put forward by Bland and Altman, is important to assess agreement parameters and whether the devices can be used interchangeably [15]. In this review, the validity of ZB heart rate variable against Polar T31 (ZB vs. Polar T31), and against gold standard criterion measure (ZB vs. ECG) yielded similar, strong to very strong correlation coefficients. However, the pair-wise agreement parameters between ZB vs. Polar T31 (two studies), and ZB vs. ECG (six studies) varied. The Johnstone et al. [1] and Johnstone et al. [24] studies, were both rated at “Very good”. However, agreement did not establish ZB agreement parameters against a gold standard criterion – ECG; instead compared ZB against Polar T31, nor provided any literature on the measurement properties of Polar T31 [1, 24]. Both studies reported wide 95% LoA. The lack of comparison against a gold standard criterion measure, and paucity of reports on the measurement properties of Polar T31, could have contributed to such wide 95% LoA. In regards to ZB vs ECG comparisons, Flanagan et al. [27] study rated at “Very good”, reported (− 2.84–2.42) 95% LoA. Similarly, Dolezal et al. [28] and Smith et al. [29] studies rated at “Good”, reported (− 0.21–0.14) and (− 3.01–0.70) 95% LoA between ZB vs ECG respectively. It is important to note that there are no thresholds to help categorized 95% LoA into excellent or poor, however, narrower 95% LoA between ZB vs ECG, is suggestive of better agreement and possible interchangeable use. On the contrary, three studies, Kim et al. [25], Rawstorn et al. [22] and Gatti et al. [26] reported somewhat wider 95% LoA in pair-wise device comparisons between ZB vs ECG. However, these studies had lower methodological scores [22, 25, 26]. Therefore, studies with higher methodological quality scores that assessed ZB vs ECG agreements, displayed narrower 95% LoA than studies with lower methodological scores.

Potential benefits of wearable technologies might include enhanced safety, better targeting of exercise to capability, better motivation and adherence. It might also allow for better progression of exercise interventions. While future studies might need to focus on the validity and utility of these devices in health promotion, monitoring or rehabilitation. The measurement studies to date are supportive of testing such applications.

The findings of our review must be considered in light of potential methodological applications. A variety of critical appraisal tools are available and the classification of quality varies across instruments. The Zephyr BioHarness measures a variety of other physiological indicators other than just heart rate, and we did not assess the reliability or validity of these other measurements. Finally, better measurement is the first step in the clinical process, and the downstream effects of using Zephyr need to be more fully investigated.

## Conclusion

Good to excellent quality evidence from ten studies suggested that the Zephyr Bioharness device can provide reliable and valid measurements of heart rate across multiple contexts, and that it displayed good agreements with gold standard measurements.

## Additional file

Additional file 1: Description of data: Quality Appraisal of a Clinical Measurement Study Tool and Interpretation Guide. (DOCX 38 kb)

## Abbreviations

CID: Clinically important difference
ICC: Intra-class correlation coefficient
LoA: Limits of agreement
MCID: Minimal clinically important difference
MID: Minimal important difference
r: Pearson’s correlation coefficients
r_s_: Spearman’s correlation coefficients
SEM: Standard error of measurement
WPM: Wearable physiological monitoring
ZB: Zephyr bioharness
