# Supplementary material for: Psychometric properties of the Zephyr bioharness device: a systematic review
Source: BMC Sports Sci Med Rehabil. 2018 Feb 21;10:6. doi: 10.1186/s13102-018-0094-4 (PMC5822593; doi:10.1186/s13102-018-0094-4)
Supplement: Supplementary file 1 — Description of data: Quality Appraisal of a Clinical Measurement Study Tool and Interpretation Guide. (DOCX 38 kb) [file 13102_2018_94_MOESM1_ESM.docx]

**Quality Appraisal of a Clinical Measurement Study**

**Interpretation Guide**

To decide which score to provide for each item on your quality checklist, read the following descriptors. Pick the descriptor that sounds *most* like the study you were evaluating with respect to a given item. If there is no documentation about any specific aspect of an item; then you must evaluate assuming that it was not done. Given the diversity in clinical measurement properties and design options, the evaluator has to make judgments using the criteria below and extend the principles to specific aspects that may not be covered in these brief exemplars. In many cases, the study will not look exactly like the descriptor so there will be some interpretation as to which level of optimal methods for clinical measurement studies have been achieved. In such cases, the evaluator can use the general approach that if this study research design and conduct is consistent with best practice (score=2); is acceptable but suboptimal (score=1); is not done/documented, substantially inadequate or inappropriate (score=0).

|  | | Descriptors | |
| --- | --- | --- | --- |
| **Study question** | | | |
| Score | |  | |
| 1 | 2 | | The authors:   - performed a thorough literature review indicating what is currently known, and not known, about the clinical measurement properties of the instruments or tests under study - presented a critical, and unbiased view of what is known about the current measurement properties - indicated how the current research question fills a gap in the current knowledge base - established a research question based on the above. |
|  | 1 | | All of the above criteria were not fulfilled, but a sound rationale was provided for the research question. |
|  | 0 | | A foundation for the current research question was not clear; and the rationale was not founded on previous literature. |
| **Study design** | | | |
| 2 | 2 | | Specific inclusion/exclusion criteria for the study were defined, that described the patients enrolled. The subjects were described in terms of health condition/demographics, key relevant outcome mediators and the recruitment context (setting). |
|  | 1 | | Some information on participants and place is provided (not all of above). For example, age/sex/diagnosis and the name or type of the practice is listed; but no additional information. |
|  | 0 | | No information on type of clinical settings or study participants is provided (other than number/mean age). |
| 3 | 2 | | Specific hypotheses or research questions are provided. The stated study purpose provides specific research questions or hypotheses that indicate which specific measurement properties will be evaluated. This should include the specific type of reliability (intra/inter-rater or test-retest) being tested or the type of validity (construct/criterion/content; longitudinal/concurrent; convergent/divergent) being tested. A prior hypothesis should describe the level of reliability expected; and for validity, expected relationships (strength of associations) or constructs. |
|  | 1 | | The types of reliability and validity being tested were apparent in the methods/title, but clear and specific research questions or hypotheses were not specified. |
|  | 0 | | Specific types of reliability or validity under evaluation were not clearly defined nor were specific hypotheses on reliability and validity stated. (“*The purpose of this study was to investigate the reliability and validity of*…” can be rated as zero if no further detail on the types of reliability and validity or the nature of specific hypotheses is stated). |
| 4 | 2 | | An appropriate scope of clinical measurement properties would be indicated by   1. A detailed focus on reliability that included multiple forms of reliability (at least two of – intra-rater, inter-rater, test retest); as well as both relative and absolute reliability (e.g., ICCs and SEM/MID or limits of agreement) 2. A detailed focus on validity that included multiple forms of validity (content (judgmental); structured (e.g., expert review/survey, qualitative interviews, ICF linking) or structural (e.g., factor analyses or Rasch), construct (known group differences; convergent/divergent associations), criterion (concurrent/predictive), responsiveness; predictive, evaluative or discriminative properties were established 3. Three or more indicators of reliability and validity were examined concurrently and provide a rich view on measurement properties. |
|  | 1 | | Two or more clinical measurement properties were evaluated, however, scope was narrow and did not meet above criteria. (e.g., internal consistency and one other indicator of validity or reliability ). |
|  | 0 | | The scope of clinical measurement properties was very narrow as indicated by a narrow evaluation of only one form of reliability or validity. |
| 5 | 2 | | Authors performed a sample size calculation and obtained their recruitment targets. Post-doc power analyses and/or confidence intervals confirm that the sample size was sufficient to define relatively precise estimates of reliability or validity. |
|  | 1 | | The authors provide an acceptable rationale for the number of subjects included in the study, but did not present specific sample size calculations or post-doc power analyses (or had a sample >100 but no justification). |
|  | 0 | | Size of the sample was not rationalized or is clearly underpowered. |
| 6 | 2 | | 90% or more of the patients enrolled for study were re-evaluated. |
|  | 1 | | 70% or more of the enrolled patients were re-evaluated. |
|  | 0 | | Less than 70% of the patients enrolled in the study were re-evaluated |
| **Measurements** | | | |
| 7 | 2 | Documentation is provided for how the studied test is performed. This includes adequate description of the measure/test and how it is administered or scored. The authors may provide or reference a published manual/article that outlines specific procedures for administration, scoring (including scoring algorithms, handling of missing data) and interpretation that included any necessary information about positioning/active participation of the client, any special equipment required, calibration of equipment if necessary, training required, cost, examiner procedures/actions. If no manual is available, then the text describes key details of procedures in sufficient detail so they could be replicated. | |
|  | 1 | The test(s) and its administration procedures are referenced; but there is inadequate description of the test procedures. | |
|  | 0 | Minimal description of test procedures without appropriate references. | |
| 8 | 2 | This item addresses the overall study procedures for administering all study measures (study measure and its comparators) in an unbiased way. Test procedures should not introduce systematic errors in the estimation of the clinical measurement properties. This includes standardized procedures for who completed or administered the measures. For self-report, this includes order of presentation, who completed at what time interval; handling of missing items. If relevant, then the paper should include how cultural literacy issues were handled (e.g., exclusion, assisted or surrogate completion). For impairment measures, procedures would include calibration of any equipment; use of consistent measurement tools and scoring, a priori exclusion of any participants likely to give invalid results/unable to complete testing (not exclusion of after enrollment); use of standardized instructions and test procedures. This can include order of administration of test and quality checking of scores. For reliability testing, the appropriate retest interval will depend on the nature of the condition; but for acute conditions it may require retesting within 48 hours; whereas chronic/stable conditions are commonly retested within 4-14 days. For estimation of clinical change, retest intervals should be ones during which a meaningful clinical change would have occurred (and from an intervention with known effectiveness). The evaluator decides overall whether this has sufficiently been addressed by the methods described. | |
|  | 1 | No obvious sources of bias in the study test protocol or how tests were performed/administered is apparent; but there were suboptimal procedures or an inadequate description of the measurement protocol to be insured control of bias or that procedures were standardized. | |
|  | 0 | No description of the overall procedures for administering study tests; OR an obvious source of bias in data collection methods. | |
| **Analyses** | | | |
| 9 | 2 | Authors clearly defined which specific analyses were conducted for each of the stated specific hypotheses/questions of the study. This may be accomplished through organization of the results under specific subheadings or by demarcating which analyses addressed specific clinical measurement properties. Data was presented for each hypothesis/research question posed. | |
|  | 1 | Data was presented that addressed each of the measurement questions posed, but authors did not link specific analyses to specific research questions or hypotheses. | |
|  | 0 | Data was not presented for every hypothesis or clinical measurement property outlined in the purposes or methods. | |
| 10 | 2 | Tests selected - Appropriate statistical tests were conducted to calculate a point estimate for clinical measurement properties. Examples are provided below; but are not exhaustive.  1. Reliability (Relative=ICCs (Shrout & Fleiss, 1979) for quantitative, Kappa (Landis & Koch, 1977) for nominal data); absolute (SEM or plot of score differences vs. average score showing mean and 2SD limit – as per Altman and Bland) (Bland & Altman, 1986; Bland & Altman, 1987)  2. Clinical relevance - minimal detectable change, clinically important difference (Jaeschke, Singer, & Guyatt, 1989; Beaton et al., 2001; Wells et al., 2001)  3. Validity  a. Validity associations - Pearson correlations for normally distributed data, Spearman rank correlations for ordinal data; or other correlations, if appropriate  b. Validity tests of significant difference - an appropriate global test like analysis of variance was used where indicated, with post-hoc tests that adjusted for multiple testing  c. Validity of items scaling/responses - Rasch analysis or item response (Baylor et al., 2011; Pallant & Tennant, 2007; Kyngdon, 2006; Cipriani, Fox, Khuder, & Boudreau, 2005; Smith, Jr., Conrad, Chang, & Piazza, 2002)  4. Responsiveness (Beaton, Bombardier, Katz, & Wright, 2001)- standardized response means or effect sizes or other recognized responsiveness indices were used. | |
|  | 1 | Appropriate statistical tests were used in some instances; but suboptimal choices were made in other analyses. | |
|  | 0 | Inappropriate use of statistical tests - incorrect tests for type of data; or a lack of analysis | |
| 11 | 2 | The study goes beyond a single statistical point estimate of a clinical measurement property and providing supporting statistical analyses that increases confidence in the findings in terms of precision of the (key) indicator; or provide an alternate form of analysis of the clinical measurement property. The evaluator decides if these analyses are appropriate and informative. For example, with reliability, at least 2 of the following would constitute appropriate and informative analysis beyond a point estimate of a reliability coefficient: 1. confidence intervals around the point estimate; 2. Comparison to appropriate, referenced benchmarks or standards; or 3. SEM or MDC. For correlations, tests of significance or confidence intervals were presented and indicators of the criterion benchmarks were provided. For studies involving cross-cultural validation, the analyses should compare multiple clinical measurement properties previously established for the measure and explain the extent to which the translated version is in accordance with these previously reported properties on the source measure. | |
|  | 1 | Either precision definition (confidence intervals) or appropriate benchmark comparison were used - NOT both. OR Some analyses were associated with indicators of precision or alternate form of analysis -but not all key indicators. | |
|  | 0 | Inappropriate use of benchmarks or confidence intervals; or indicators of precision or alternate form are absent | |
| **Recommendations** | | | |
| 12 | 2 | Authors made specific conclusions and clinical measurement recommendations that were clearly related to each hypotheses/question posed in the study and that were supported by the data presented. Ideal recommendations would state the estimated status of the clinical measurement property, the confidence in the estimate and the context for which those apply. To achieve a 2, the conclusion must be specific; and conclusions cannot overstate the clinical measurement properties observed the study; nor ignore suboptimal measurement properties found. | |
|  | 1 | Authors made conclusions and clinical measurement recommendations that were basically true (supported by study data); but vague. That is, they do not specify the extent, confidence or context of the findings. (The measure is “reliable and valid ”) OR authors made specific clinical measurement recommendations; but for only some of the study hypotheses. | |
|  | 0 | Authors did not make conclusions about clinical measurement; OR made recommendations that were in contradiction to the actual data presented | |

© MacDermid 2011

**Quality Appraisal for Clinical Measurement Research Reports**

**Evaluation Form**

Authors: _____________________________ Year: ___________________ Rater: ____

*Use this form to rate the quality of a clinical measurement study. To decide which score to provide for each item on your quality checklist, pick the descriptor that sounds most like what was reported in the study you are evaluating. Items rank descriptors are provided in the guide. (Forms and guides to extract study data for evidence synthesis are available from developer at macderj@mcmaster.ca)*

| **Evaluation criteria** | **Score** | | |
| --- | --- | --- | --- |
| Study question | 2 | 1 | 0 |
| 1. Was the relevant background work cited to define what is currently known about the measurement properties of measures under study, and the potential contributions of the current research question to informing that knowledge base? |  |  |  |
| Study Design |  |  |  |
| 2. Were appropriate inclusion/exclusion criteria defined? |  |  |  |
| 3. Were specific clinical measurement questions/hypotheses identified? |  |  |  |
| 4. Was an appropriate scope of measurement properties considered? |  |  |  |
| 5. Was an appropriate sample size used? |  |  |  |
| 6. Was appropriate retention/follow-up obtained? (for studies involving retesting; otherwise n/a) |  |  |  |
| Measurements |  |  |  |
| 7. Were specific descriptions provided of the measure under study and the method(s) used to administer it? |  |  |  |
| 8. Were standardized procedures used to administer all study measures in a manner that minimized potential sources of error/bias (including the study measure and its comparators)? |  |  |  |
| **Analyses** |  |  |  |
| 9. Were analyses conducted for each specific hypothesis or purpose? |  |  |  |
| 10. Were appropriate statistical tests performed to obtain point estimates of the measurement properties? |  |  |  |
| 11. Were appropriate ancillary analyses done to quantify the confidence in the estimates of the clinical measurement property (Precision/Confidence intervals; benchmark comparisons/ROC curves, alternate forms of analysis like SEM/MID, etc.)? |  |  |  |
| Recommendations |  |  |  |
| 12. Were clear, specific and accurate conclusions made about the clinical measurement properties; that were associated with appropriate clinical measurement recommendations and supported by the study objectives, analysis and results? |  |  |  |
| **Subtotals** (of columns 1 and 2) |  |  |  |
| **Total score** (sum of subtotals/24*100);  if for a specific paper or topic an item is deemed inappropriate then you can sum of items/2*number of items *100 |  |  |  |

© MacDermid 2011

Reference List

Baylor, C., Hula, W., Donovan, N. J., Doyle, P. J., Kendall, D., & Yorkston, K. (2011). An introduction to item response theory and Rasch models for speech-language pathologists. *Am.J.Speech Lang Pathol., 20,* 243-259.

Beaton, D. E., Bombardier, C., Katz, J. N., & Wright, J. G. (2001). A taxonomy for responsiveness. *J.Clin.Epidemiol., 54,* 1204-1217.

Beaton, D. E., Bombardier, C., Katz, J. N., Wright, J. G., Wells, G., Boers, M. et al. (2001). Looking for important change/differences in studies of responsiveness. OMERACT MCID Working Group. Outcome Measures in Rheumatology. Minimal Clinically Important Difference. *J.Rheumatol., 28,* 400-405.

Bland, J. M. & Altman, D. G. (1987). Statistical methods for assessing agreement between measurements. Biochem Clin 11, 399-404.

Bland, J. M. & Altman, D. J. (1986). Statistical methods for assessing agreement between two methods of clinical measurement. *Lancet, 1,* 307-310.

Cipriani, D., Fox, C., Khuder, S., & Boudreau, N. (2005). Comparing Rasch analyses probability estimates to sensitivity, specificity and likelihood ratios when examining the utility of medical diagnostic tests. *J.Appl.Meas., 6,* 180-201.

Jaeschke, R., Singer, J., & Guyatt, G. H. (1989). Measurement of health status. Ascertaining the minimal clinically important difference. *Control Clin.Trials, 10,* 407-415.

Kyngdon, A. (2006). An introduction to the theory of unidimensional unfolding. *J.Appl.Meas., 7,* 260-277.

Landis, J. R. & Koch, G. G. (1977). The measurement of observer agreement for categorical data. *Biometrics, 33,* 159-174.

Pallant, J. F. & Tennant, A. (2007). An introduction to the Rasch measurement model: an example using the Hospital Anxiety and Depression Scale (HADS). *Br.J.Clin.Psychol., 46,* 1-18.

Shrout, P. E. & Fleiss, J. L. (1979). Intraclass correlations: Uses in assessing rater reliability. *Psychological Bulletin, 86,* 420-428.

Smith, E. V., Jr., Conrad, K. M., Chang, K., & Piazza, J. (2002). An introduction to Rasch measurement for scale development and person assessment. *J.Nurs.Meas., 10,* 189-206.

Wells, G., Beaton, D., Shea, B., Boers, M., Simon, L., Strand, V. et al. (2001). Minimal clinically important differences: review of methods. *J.Rheumatol., 28,* 406-412.
